# Current Chemotherapeutic Management of Patients with Gestational Trophoblastic Neoplasia

**DOI:** 10.1155/2011/806256

**Published:** 2011-05-11

**Authors:** Taymaa May, Donald P. Goldstein, Ross S. Berkowitz

**Affiliations:** Brigham and Women's Hospital and Dana-Farber Cancer Institute, Department of Obstetrics and Gynecology, Division of Gynecologic Oncology, New England Trophoblastic Disease Center, Harvard Medical School, Boston, MA 02115, USA

## Abstract

Gestational trophoblastic neoplasia (GTN) describes a heterogeneous group of interrelated lesions that arise from abnormal proliferation of placental trophoblasts. GTN lesions are histologically distinct, malignant lesions that include invasive hydatidiform mole, choriocarcinoma, placental site trophoblastic tumor (PSTT) and epithelioid trophoblastic tumor (ETT). GTN tumors are generally highly responsive to chemotherapy. Early stage GTN disease is often cured with single-agent chemotherapy. In contrast, advanced stage disease requires multiagent combination chemotherapeutic regimens to achieve a cure. Various adjuvant surgical procedures can be helpful to treat women with GTN. Patients require careful followup after completing treatment and recurrent disease should be aggressively managed. Women with a history of GTN are at increased risk of subsequent GTN, hence future pregnancies require careful monitoring to ensure normal gestational development. This article will review the workup, management and followup of women with all stages of GTN as well as with recurrent disease.

## 1. Introduction

Gestational trophoblastic neoplasia (GTN) are malignant lesions that arise from abnormal proliferation of placental trophoblast. The pathologic conditions that make up this entity include invasive partial and complete hydatidiform mole, choriocarcinoma, placental site trophoblastic tumor (PSTT), and epithelioid trophoblastic tumor (ETT). GTN often arises after molar pregnancies but can also occur after any gestation including miscarriages and term pregnancies. In the United States, hydatidiform moles are observed in approximately 1/600 therapeutic abortions and 1/1000–2000 pregnancies [1, 2]. Fortunately, these malignancies are highly susceptible to chemotherapy and it is often possible to achieve cure while preserving the woman's reproductive function [3–7]. This article will review the current chemotherapeutic management of patients with GTN.

## 2. Workup of Patients with GTN

Women newly diagnosed with GTN require a thorough evaluation of the extent of their disease such that the appropriate treatment can be selected. This evaluation includes a history and physical exam, serum quantitative hCG level, a complete blood count, and hepatic and renal function tests. 

A pelvic ultrasound is often useful to detect the extent of uterine involvement and may identify patients who are at risk for uterine perforation or who would benefit from a hysterectomy to reduce tumor burden [7, 8]. A chest X-ray should be obtained to evaluate lung metastasis. If this is negative, a chest computed tomography (CT) scan may be performed since it may detect micrometastases in 40% of patients with a negative chest X-ray [9, 10]. Additional imaging can be omitted in asymptomatic patients with a negative chest CT given that distant metastases are unlikely in the absence of lung metastases. Conversely, abdominal and brain imaging are an essential part of the workup in patients with metastases to the vagina or to the lungs and in patients with a histological diagnosis of choriocarcinoma. Furthermore, an elevated cerebral spinal fluid/plasma hCG ratio may suggest cerebral involvement [11, 12]. Additional imaging, such as 18-fluorodeoxyglucose positron emission tomography (FDG-PET), may be useful to accurately outline sites of metabolically active or viable metastases and help determine the potential for tumor resectability [13]. If a patient has a drug-resistant disease, a PET scan can help determine if a persistent radiographic finding has viable, active tumor.

## 3. GTN after a Molar Gestation

The risk of developing GTN after a complete hydatidiform mole (CHM) and a partial hydatidiform mole (PHM) is 15–20% and 1–4%, respectively [14]. 

In accordance with the International Federation of Gynecology and Obstetrics (FIGO), GTN is diagnosed after a molar gestation if any of the following is observed [15]:

1. four values or more of hCG plateau over at least three weeks (days 1, 7, 14, and 21),
2. a rise in hCG of 10% or greater for three or more values over at least two weeks (days 1, 7, and 14),
3. the presence of histologic choriocarcinoma,
4. persistence of hCG six months after molar evacuation.

CHM is well recognized to have the potential for local invasion and distant spread. Following evacuation of a CHM, local uterine invasion occurs in approximately 15% and metastases is observed in 4% of patients [14]. Interestingly, patients with CHM who present with excessive uterine size and markedly elevated hCG levels (>100,000 mIU/mL) develop GTN in 40–50% of cases and are considered high risk [14]. Postmolar, locally-invasive GTN commonly presents with irregular vaginal bleeding. In certain cases, the invasive tumor may erode into the uterine vessels leading to significant vaginal hemorrhage. In other instances, it can perforate through the myometrium leading to intraperitoneal hemorrhage. In addition, a necrotic intrauterine tumor may serve as a focus for infection.

## 4. FIGO Staging and Prognostic Score

When reporting GTN data, it is useful to use both the FIGO anatomic staging system and prognostic scoring system [15] (Tables 1 and 2). A FIGO score of 6 or less indicates low-risk GTN whereas a score of 7 or more identifies high-risk disease. High-risk GTN has increased resistance to single-agent chemotherapeutics, increased risk of recurrence, and generally requires combination chemotherapy to achieve remission.

## 5. Metastatic GTN

Metastatic GTN occurs in 4% of patients after evacuation of CHM and infrequently after other pregnancies [14]. The most common metastatic sites are the lung (80%), vagina (30%), brain (10%), and liver (10%) [16]. Trophoblastic tumors are perfused by fragile vessels and, as a result, metastases are often hemorrhagic. Biopsy of metastases is neither necessary nor recommended due to the risk of hemorrhage. Patients may present with signs and symptoms of bleeding from metastases such as hemoptysis, intraperitoneal bleeding, or acute neurologic deficits. Cerebral and hepatic metastases are uncommon unless there is concurrent involvement of the lungs and/or vagina. 

Patients with pulmonary metastases commonly have asymptomatic lesions on chest radiography or they may present with dyspnea, chest pain, cough, or hemoptysis. Trophoblastic emboli may cause pulmonary arterial occlusion and lead to rightheart strain and pulmonary hypertension [17]. This may lead to a false diagnosis of primary pulmonary disease especially if the antecedent pregnancy is remote and the gynecologic symptoms are minimal or absent. It is, therefore, imperative to consider GTN in any woman in the reproductive age group with unexplained systemic or pulmonary symptoms. Interestingly, 40% of patients with presumed nonmetastatic disease have occult pulmonary nodules on CT scan [9, 10]. Given that the FIGO staging system includes findings on CXR, but not CT scan, these patients will be classified as stage I; however, their management might be influenced by the CT findings.

The vagina is the second most common site of metastasis and is involved in 30% of metastatic cases. Patients often present with irregular vaginal bleeding or purulent vaginal discharge. Vaginal examination often reveals vascular lesions, most commonly located in the suburethral region or at the vaginal fornices. Given the high vascularity of these lesions, they should not be biopsied since this can lead to significant hemorrhage.

A small portion of women with GTN will have cerebral metastases. Cerebral involvement can cause increased intracranial pressure and intracerebral bleeding, which often leads to neurological symptoms. As such, most women with brain involvement have clear neurological symptoms including nausea, vomiting, headache, seizures, slurred speech, visual disturbances, or hemiparesis [18–22]. Neurological symptoms were observed in patients with brain metastasis in 20 of 23 patients (87%) by Bakri et al., 66 of 69 patients (96%) by Athanassiou et al., and in all 34 patients (100%) by Liu et al. [19, 21, 22].

Infrequently, women with GTN will have liver metastases. The majority of these patients will have disease involving other organ systems such as the lungs, vagina, and other distant sites. Interestingly, these patients uncommonly present with symptoms related to hepatic involvement but rather with symptoms related to involvement of the other sites. Bakri et al. noted that only 5 of 19 patients (26%) with liver metastases presented with liver-related complaints, such as jaundice, intraabdominal bleeding, or epigastric pain [23].

## 6. Treatment of Low-Risk GTN

Patients with stage I and low-risk stage II and III GTN (FIGO prognostic score of ≤6) generally respond well to single-agent chemotherapy. A repeat dilation and curettage is usually not indicated as it may increase risks of bleeding and infection without decreasing the need of adjuvant chemotherapy. The most commonly used agents for low-risk GTN are sequential methotrexate (MTX) and actinomycin-D (ACT-D). At the New England Trophoblastic Disease Center (NETDC), we use MTX as a first-line agent given its lower side effect profile as compared to ACT-D [24]. MTX causes less nausea, less vomiting, and no alopecia. We believe that ACT-D can be used as a first-line agent in patients with hepatic dysfunction or who have a known adverse reaction to MTX. 

Fortunately, single-agent chemotherapy can be very effective in treating low-risk GTN. At our center, 632 women with low-risk GTN were treated with single-agent chemotherapy between 1965 and 2006. Complete remission was achieved in 419 of 502 patients (83.5%) with stage I GTN, 16 of 20 patients (80%) with low-risk, stage II disease, and 90 of 110 patients (81.8%) with low-risk, stage III GTN. After administering the first course of chemotherapy, it is our practice to closely monitor patients' hCG level. Further chemotherapy is given if the hCG value fails to decline by 1 log within 18 days of the first treatment, if the hCG level plateaus for more than 3 consecutive weekly values, or if the hCG level begins to rise. 

Over the past several decades, multiple treatment protocols were effectively used in treating low-risk GTN [25–33]. Nonetheless, there has never been a trial that compared the effectiveness of all the regimens. At the NETDC, we administer MTX in an eight-day treatment regimen consisting of four administrations of MTX given at 1 mg/kg every other day with folinic acid 0.1 mg/kg given on intervening days. When we compared this protocol to one where patients received 100 mg/m^2^ intravenous (IV) MTX bolus followed by 200 mg/m^2^ IV MTX infused over 12 hours followed by folinic acid, the eight-day regimen was associated with a higher remission rate in patients with low-risk GTN [34]. 

An important phase III randomized trial examining MTX and ACT-D in the treatment of low-risk GTN was published by the Gynecologic Oncology Group (GOG) in 2011 [35]. Two hundred sixteen patients were randomized to receive either biweekly ACT-D 1.25 mg/m^2^ IV or weekly MTX 30 mg/m^2^ intramuscularly (IM). The remission rate was 58% in the MTX arm and 73% in the ACT-D arm. These results suggest that ACT-D is superior to the weekly MTX regimen in treating low-risk GTN. However, before recommending pulse ACT-D as the primary modality in the treatment of patients with low-risk GTN it is important to be aware of the potential for significant toxicity of this regimen as compared to those with MTX. Furthermore, all patients with low-risk disease in the Osborne series ultimately achieved remission regardless of their initial response. Therefore, in order to reach definitive conclusions regarding the true comparative effectiveness of these agents, it would be prudent to compare the biweekly ACT-D regimen to the more commonly used 5-day or 8-day MTX regimens which offer a high initial remission rate with minimal toxicity. 

In women who no longer desire fertility, a hysterectomy may be considered in stage I disease to decrease exposure to multiple doses of chemotherapeutic agents. In this treatment strategy, the patient receives one course of single-agent chemotherapy at the time of surgery. This is imperative to treat occult metastases that may be present at the time of surgery, to reduce the likelihood of disseminating viable tumor cells during surgery, and to maintain a cytotoxic level of chemotherapy in the blood and tissues in case viable tumor cells are disseminated during surgery. At our center, 32 patients with stage I GTN were treated with a hysterectomy and perioperative chemotherapy. None of the patients experienced intraoperative or perioperative complications, none required additional adjuvant therapy, and all patients achieved complete remission. This suggests that administration of chemotherapy at the time of hysterectomy is a safe and effective treatment strategy for selected women with stage I GTN. 

Despite the excellent effectiveness of MTX and ACT-D in treating low-risk GTN, some patients experience resistance to both agents. Recent data from Charing Cross Hospital indicate that patients with a low-risk FIGO score but with an hCG value exceeding 100,000 mIU/mL frequently require combination chemotherapy [36]. These women are often treated with MAC (MTX, ACT-D, and Cyclophosphamide) or EMACO (Etoposide, MTX, ACT-D, Cyclophosphamide, and Vincristine). MAC is preferred as the initial combination chemotherapy regimen since etoposide, which is a component of EMACO, is associated with an increased risk for secondary malignancies. Studies have shown that patients treated with more than 2 g/m^2^ of etoposide, had a relative risk of 16.6 for developing leukemia, 5.8 for breast cancer, 4.6 for colon cancer, and 3.4 for melanoma [37].

In addition to medical therapy, surgical management may be useful in cases where GTN continues to be resistant to combination chemotherapy. Preoperative imaging studies such as ultrasound, MRI, arteriography, and positron emission tomography (PET) scans may be helpful in identifying the site of residual tumor and can, therefore, facilitate surgical planning [13, 38]. If a large tumor volume is identified within the uterus, a hysterectomy may be performed to remove the focus of drug resistance. If the woman desires to retain her fertility, local uterine resection of the tumor mass can be attempted. This management option should be reserved for carefully selected patients. Women should be counseled about the potential future obstetrical risks associated with this procedure, including uterine rupture [39]. Two patients at the NETDC were successfully treated in this manner, and one patient went on to carry a pregnancy to term and was delivered by cesarean section. Allison et al. reported a patient with persistent GTN who underwent fertility-sparing surgery and went on to have a successful pregnancy [39]. Similarly, Behtash et al. described a patient with choriocarcinoma and a perforated uterus who was treated with localized uterine resection and subsequently had an uncomplicated pregnancy [40]. 

*Table 3 summarizes the NETDC's protocol for treating stage I GTN, and Table 4 summarizes the NETDC's protocol for treating low-risk, stage II and III disease.*

## 7. Treatment of High-Risk, Stage II and III GTN

Patients with stage II or III GTN and an FIGO prognostic score ≥7 have a high-risk disease and are unlikely to be cured with single-agent therapy. Therefore, they should be treated with combination chemotherapy [41]. Importantly, MAC is inadequate as primary treatment for high-risk, metastatic GTN as it induces remission in only half the patients [42–44]. At the NETDC, EMACO is the first-line regimen used to treat high-risk GTN since it has the best effectiveness-to-toxicity ratio. At our center, combination chemotherapy induced complete remission in 6 of 6 patients (100%) with high-risk stage II GTN and in 36 of 37 patients (97.3%) with high-risk stage III GTN. At other centers, Bower et al. and Bolis et al. reported that EMACO induced remission in 86% and 76% of patients with high-risk metastatic GTN, respectively [45, 46].

Although EMACO is the most commonly used combination chemotherapy, other regimens have been used in the management of high-risk GTN. In a retrospective analysis of four chemotherapeutic regimens, Kim et al. compared the effectiveness of MFA (MTX, folinic acid, ACT-D), MAC, CHAMOCA (cyclophosphamide, hydroxycarbamide, doxorubicin, ACT-D, MTX, melphalan, and vincristine) and EMACO. They reported remission rates of 63%, 68%, 71%, and 91%, respectively [47]. These results support EMACO's effectiveness as primary therapy for patients with high-risk disease. Combination chemotherapy is often administered at two- to three-week intervals and timely administration is essential. Unnecessary treatment delays and dose reductions should be avoided as they may lead to tumor resistance and treatment failure. Patients receiving combination chemotherapy should have serial hCG measurements. After the first undetectable hCG level, 2 to 4 additional chemotherapy courses are administered to reduce the risk of relapse [48–50]. 

Patients with disease resistant to EMACO can be treated using EMAEP—a regimen that substitutes cyclophosphamide and vincristine on day 8 with cisplatin and etoposide [45, 51]. In 21 patients with disease resistant to EMACO, 16 (76%) were successfully treated with EMAEP, either alone or with surgery [45]. 

Other regimens have also been effective in treating high-risk refractory GTN including BEP (bleomycin, etoposide, cisplatin), ICE (ifosfamide, carboplatin, etoposide), and VIP (etoposide, ifosfamide, cisplatin) [52–55]. In a case report, Willemse et al. describe a 53-year-old woman with metastatic GTN who did not respond to initial treatment with a combination of MTX, ACT-D, and chlorambucil and who was subsequently treated with BEP [52]. Complete remission was achieved; however, the patient suffered from moderate, transient bleomycin pneumonitis. 

Piamsomboon et al. reported a patient who developed brain metastasis while on EMACO. The patient was successfully treated with a low-dose salvage ICE regimen [53]. Furthermore, a phase I-II trial was conducted by Lotz et al. to assess the toxicity and efficacy of the ICE regimen combined with autologous bone marrow transplantation in germ cell tumors and metastatic GTN [54]. Thirty-nine patients were studied, including 5 with drug-resistant GTN. The overall response rate was 46%, including a complete response rate of 35%. Among the 5 patients with GTN, the complete response rate was 40% (2/5) and there were no partial responses. Importantly, several patients reported serious side effects, which were primarily renal toxicity and enterocolitis. It is not clear how many patients with GTN suffered from major side effects given that the toxicity incidence was not stratified according the patient's diagnosis. In addition, 7 of 39 patients (18%) died of therapy-related causes, including one patient with GTN who developed refractory thrombocytopenia and died of cerebral hemorrhage. Hence, although the ICE regimen appears to overcome drug resistance in highly refractory germ cell tumors and GTN, it was associated with significant morbidity and mortality [54].

To compare various combination chemotherapy regimens, Lurain and Nejad evaluated 26 patients with relapsed GTN [55]. Sixteen patients were initially treated with MTX or ACT-D and 10 with EMACO. Of the 16 patients who resisted initial MTX or ACT-D therapy, 10 (63%) achieved complete remission after secondary therapy consisting mainly of platinum and etoposide combinations. Eight patients were treated with BEP, 1 with VIP, and 1 with ICE. Of the 10 patients with resistance to EMACO, 9 (90%) achieved complete remission with second-line chemotherapy. Three patients were treated with EMAEP and 6 with BEP. The authors, therefore, concluded that patients with persistent or recurrent GTN should be treated with drug combinations employing both a platinum agent and etoposide with or without bleomycin or ifosfamide [55]. 

In some women, chemotherapy alone may not successfully treat GTN, and they may benefit from adjuvant surgical excision of the chemoresistant tumor [7]. Clark et al. reported that 25 of 33 women (76%) with chemoresistant uterine tumor achieved complete remission with hysterectomy [56]. An additional benefit to surgical treatment is that it may reduce tumor mass and may decrease the dose and length of administration of chemotherapy. This was highlighted in a report by Hammond et al. stating that patients who underwent hysterectomy had shorter hospitalization and a briefer chemotherapy treatment course [7]. 

An interesting procedure in the management of stage III GTN is thoracotomy, which has a limited yet important role in the management of lung metastases. Thoracotomy can be useful to establish the diagnosis of GTN if the diagnosis cannot be clarified using less invasive measures. In addition, in patients with persistent, viable pulmonary nodules that are resistant to chemotherapy, pulmonary resection may be curative [57]. Tomoda et al. reported on 19 patients with chemoresistant GTN that were treated with adjuvant thoracotomy [58]. Based on their experience, they proposed the following criteria to predict successful pulmonary resection: (a) patient is a good surgical candidate, (b) primary malignancy is controlled, (c) no evidence of other metastatic sites, (d) pulmonary metastasis is limited to one lung, and (e) hCG level is <1,000 mIU/mL. In their series, complete remission was achieved in 14 of 15 patients (93%) who met all five criteria but in zero of 4 patients (0%) who met only 4 or less of the criteria [58]. Similarly, Fleming et al. reported that ten of eleven carefully selected patients (90.9%) with drug-resistant pulmonary metastases achieved remission following resection of the pulmonary tumor [59]. Notably, an undetectable hCG level within 2 weeks of resection of a solitary pulmonary nodule is highly predictive of a favorable outcome [60–64]. Although pulmonary resection can be useful in selected cases, it is important to note that thoracotomy is seldom necessary and that most lung lesions can be successfully treated with chemotherapy. 

*Table 4 summarizes the NETDC's protocol for managing high-risk, stage II and III GTN.*

## 8. Treatment of Stage IV GTN

Strikingly, prior to 1975, survival of patients with stage IV GTN at the NETDC was only 30%. Since then, the survival of these women has risen dramatically to 80% largely due to the introduction of early, concentrated, multiagent chemotherapy. Hence, all patients with stage IV disease at the NETDC are now treated with primary, intensive, combination chemotherapy. The most common combination chemotherapy we use to treat stage IV disease is EMACO. Frequently, radiation treatment and surgical interventions are also used as adjuvant therapy in this setting. 

Women with cerebral metastases may pose specific treatment challenges. The first modification to the EMACO regimen in these patients is to increase the MTX dose on day 1 to 1 gm/m^2^ to ensure adequate coverage of brain tissue [65, 66]. If resistance to EMACO is encountered, EMAEP can be substituted effectively. The use of adjuvant brain irradiation is controversial. Yordan et al. reported that mortality due to central nervous system involvement was 44% (11 of 25) in patients treated with chemotherapy alone whereas the mortality was zero in 18 patients treated with chemotherapy and brain irradiation [67]. In contrast, data from the Charing Cross Hospital reported that 30 of 35 patients (86%) with cerebral lesions achieved sustained remission with intensive combination chemotherapy, which included high-dose intravenous and intrathecal MTX, without brain irradiation [65]. At the NETDC, we institute adjuvant radiation therapy in all patients with cerebral metastases. In addition, peripheral, solitary cerebral lesions may be amenable to surgical resection [68]. Evans et al. reported complete remission in 3 of 4 patients (75%) who underwent craniotomy to relieve intracranial pressure, and in 2 of 3 patients (66%) undergoing craniotomy for resection of chemoresistant tumor [68]. Similarly, Athanassiou et al. reported that 4 of 5 patients (80%) undergoing craniotomy for acute intracranial complications were ultimately cured [21]. Importantly, the majority of patients with treated cerebral metastases have no residual neurologic deficits. 

Women with stage IV GTN with hepatic involvement are often successfully treated with chemotherapy alone as described by Wong et al. and Bakri et al., where 9 of 10 patients (90%) and 5 of 8 patients (62.5%), respectively, achieved complete remission with primary intensive combination chemotherapy [23, 69]. 

*Table 5 outlines the NETDC protocol for managing stage IV GTN.*

## 9. Treatment of PSTT and ETT

While hysterectomy is reserved for selected patients with GTN, it should be considered as a first-line treatment strategy in women with stage I PSTT and ETT given that these are relatively chemoresistant neoplasms. Women with stage I PSTT or ETT are often effectively treated with surgery alone. Patients with metastatic PSTT may still achieve remission with intensive combination chemotherapy after surgical intervention, particularly when they are diagnosed within 4 years of the antecedent pregnancy [70, 71]. ETT is the rarest variety of GTN, hence there is limited data on the optimal chemotherapy to treat patients with advanced-stage disease [72].

## 10. Management of Complications from GTN

Women with GTN may present with complications related to their disease and this may necessitate urgent management including surgical interventions. Not infrequently, hysterectomy may become necessary to control profuse uterine hemorrhage or to remove an infected tumor focus [54]. In a retrospective study by Cagayan and Magallanes, 134 women in the Philippines with GTN were studied [73]. Interestingly, 13 of 134 patients (9%) required a hysterectomy for profuse vaginal bleeding, whereas 31 of 134 patients (24%) underwent urgent hysterectomy for uterine rupture. In patients who are hemodynamically stable, angiographic uterine embolization can be effective in the management of uterine bleeding, especially in women who wish to retain their fertility [74].

Vaginal metastases are often highly vascular and friable and may bleed profusely. Yingna et al. reported that 18 of 51 patients (35.3%) with vaginal metastases presented with vaginal hemorrhage [75]. Bleeding is often controlled by packing the vagina. However, other modalities may become necessary such as wide local excision of the lesions or angiographic embolization of the hypogastric vessels. Yingna et al. reported that vaginal bleeding was successfully controlled with vaginal packing in 16 of 18 patients (88%) and with angiographic embolization in the remaining 2 patients [75]. 

The management of complications from hepatic metastases can be particularly challenging in patients with advanced GTN. In rare instances, hepatic resection may be required to control bleeding. Grumbine et al. reported on the use of selective hepatic arterial occlusion and concurrent combination chemotherapy in a patient with bleeding liver metastases who ultimately attained complete remission [76].

## 11. Followup of Patients with GTN

All patients with GTN should be followed with weekly serum quantitative hCG levels until normal for 3 consecutive weeks, then monthly for 12 months. The monthly followup period is extended to 24 months in patients with stage IV disease given the increased risk of late recurrence in this patient population. It is important to note that GTN often generates fragmented or degraded hCG molecules. As such, trophoblastic disease samples may contain high proportions of free *β*-hCG, nicked hCG, as well as *β*-core fragments [77, 78]. Hence, it is imperative to use an hCG assay that detects hCG metabolites and fragments when monitoring patients with history of GTN [79–82].

## 12. Patients with Persistent Low hCG Levels

In a subset of patients with GTN, the hCG may plateau at very low levels for several weeks or months. Metastatic workup in these patients is often negative, and they are characterized as having quiescent GTN. Interestingly, the hCG form that is primarily present in these cases is the nonhyperglycosylated form [82–85]. Provided their hCG remains stable, these patients do not require any additional chemotherapy and are generally chemoresistant. However, sustained hCG followup is essential as the hCG values may begin to rise in 6–10% of women with quiescent GTN after a prolonged period of stability. When the hCG elevates, a higher percentage of the hCG becomes hyperglycosylated and this is indicative of disease recurrence, at which point chemotherapy should be instituted. 

An intriguing subset of women may present with elevated hCG levels but without a clear antecedent pregnancy and without a progressive rise in their hCG value. This phenomenon may be due to circulating heterophilic hCG antibodies and is termed phantom hCG or phantom GTN [79]. Negative urine hCG levels will distinguish false positive phantom serum hCG from true positive serum hCG and will confirm the diagnosis of phantom GTN.

In some instances, the hCG assays may have cross-reactivity with luteinizing hormone (LH), which can lead to false positive results if the patient's LH levels are elevated. This is particularly relevant in women with ovarian impairment secondary to combination chemotherapy or to peri- or postmenopausal status. It is, therefore, advisable to treat women receiving combination chemotherapy with the oral contraceptive pill to suppress LH levels and avoid false positive hCG results. 

Furthermore, peri- and postmenopausal women may have persistent low levels of hCG that is pituitary in origin. Pituitary hCG is physiologically produced with increasing menopausal production of luteinizing hormone, owing to the decreased production of estrogen and the suppression of progesterone [86, 87]. To distinguish this phenomenon from hCG secondary to trophoblastic disease, women can be treated with estrogen, which would suppress pituitary hCG but would not alter GTN produced hCG.

## 13. Recurrent GTN

Women with history of GTN have a potential risk of disease recurrence that is largely dependent on their initial stage. Mutch et al. reported recurrence rates of 2% in patients with nonmetastatic GTN, 4% in patients with low-risk, metastatic GTN, and 13% in patients with high-risk, metastatic disease [88]. At the NETDC, the reported recurrence rates were 2.9% in patients with stage I disease, 8.3% in stage II, 4.2% in stage III, and 9.1% in patients with stage IV GTN [89]. The mean time at our center from the last undetectable hCG level to documented recurrence was 6 months, irrespective of the FIGO stage. Similarly, Ngan et al. reported that the median recurrence time in women from Hong Kong with GTN was 6.5 months [90]. The risk of recurrence increased in patients with large initial tumor burden and in patients who defaulted on potential treatment or who did not comply with followup [90].

Fortunately, recurrent GTN is often curable. At the NETDC, all patients with recurrent stage I, II, and III disease were cured with chemotherapy. Two patients with recurrent stage IV GTN succumbed to their disease and the remainder of patients with recurrent stage IV GTN were ultimately cured using intensive combination chemotherapy.

## 14. Subsequent Pregnancies

After a patient experiences a molar pregnancy, her risk of developing a subsequent molar pregnancy increases from a baseline of 1/1000 to 1/100 [91]. It is, therefore, prudent to obtain a serum hCG value and a pelvic ultrasound early in the first trimester of all subsequent pregnancies to confirm normal gestational development. In addition, hCG levels should be measured 6 weeks after the completion of each future pregnancy to exclude the presence of occult trophoblastic disease.

The secondary infertility rate in women who were previously treated with chemotherapy for GTN is approximately 7% [92–99]. As such, the majority of women who are interested in conceiving after completion of GTN treatment will be successful. The reproductive outcomes of 2,657 women who were previously treated using chemotherapy for GTN at 9 centers, including the NETDC, were described in multiple reports [92–99]. The majority of pregnancies achieved live births; with 2,038 (76.7%) term births, 71 (5.3%) premature births, 34 (1.3%) stillbirths and 378 (14.2%) spontaneous miscarriages. Notably, 37 infants (1.8%) were born with congenital malformations. This rate is comparable to the general population and is reassuring that chemotherapeutic regimens used in the treatment of GTN do not appear to increase the rate of congenital anomalies in subsequent pregnancies. Furthermore, Woolas et al. noted no differences in conception rates or pregnancy outcomes between women treated with single-agent MTX and those receiving combination chemotherapy [99]. 

At the NETDC, we recommend a followup period of 12 months for women with stage I, II, and III GTN and of 24 months for stage IV disease, during which the patient is advised not to conceive. This is not only crucial to the appropriate interpretation of the hCG levels but also to the well-being of the subsequent gestation. Matsui et al. reported that pregnancies within 6 months of treatment completion are at increased risk for spontaneous miscarriages, stillbirths, and repeat moles [100].

## 15. Novel Treatments in Patients with EMACO Resistance

Patients with disease resistant to current chemotherapeutic protocols pose a significant treatment challenge and efforts continue to identify new effective agents to treat resistant GTN. Osborne et al. described a novel, 3-drug doublet regimen, consisting of paclitaxel, etoposide, and cisplatin (TP/TE) that induced complete remission in two patients with relapsed high-risk GTN [101]. Wang et al. further studied this regimen in 16 patients with chemoresistant disease, including 6 patients previously treated with platinum-based chemotherapy. Three of 16 patients (19%) achieved a complete response and 5 of 16 (31%) a partial response. In addition, the TP/TE protocol was well tolerated, with only one patient discontinuing therapy because of toxic effects. Thus, the TP/TE regimen appears effective in treating refractory GTN and may be better tolerated by patients than EMAEP [102]. Consequently, the International Society for the Study of Trophoblastic Disease (ISSTD) has proposed a randomized trial comparing TP/TE to EMAEP in patients with recurrent GTN after treatment with EMACO. 

Wan et al. described 100% efficacy of a floxuridine- (FUDR-) containing treatment when given to 21 patients with drug-resistant GTN [103]. Matsui et al. found that 5-FU in combination with ACT-D may be used as salvage therapy and induced complete remission in 9 of 11 (82%) patients with drug-resistant GTN [104]. To treat drug-resistant GTN, a combination of cisplatin, vinblastine, and bleomycin (PVB) has been studied by Gordon et al., DuBeshter et al. and Azab et al. who reported that complete remission was achieved in 2 of 11 patients (18%), 4 of 7 patients (57%), and 5 of 8 patients (62%), respectively [105–107]. Ifosfamide and paclitaxel are sometimes used to treat GTN; however, further studies are needed to confirm their potential role as either primary or secondary treatment agents [108, 109].

An interesting potential treatment avenue for women with refractory GTN is the use of autologous bone marrow transplantation or stem-cell support concurrent with high-dose chemotherapy. Although complete remission has been reported in selected cases, the proper role of these measures in the treatment of GTN is yet to be defined [110, 111].

## 16. Conclusion

Significant progress has been made over the past decades in the diagnosis and management of women with GTN. GTN is a highly curable disease that can be effectively managed with single- or multiagent chemotherapy. Nonetheless, some women succumb from GTN primarily due to late presentation, delayed diagnosis of primary or recurrent disease, or drug resistance. Therefore, educating primary care physicians and gynecologists about the signs and symptoms of GTN is essential in decreasing adverse outcomes related to this disease. In addition, careful followup of all women with GTN is important to ensure that recurrence is detected promptly, at a time when it is curable. Moreover, the discovery of novel therapeutics may decrease drug toxicity, enhance treatment efficacy, and improve the management of women with chemoresistant disease. 

Lastly, it is well established that the diagnosis of GTN may have a significant emotional impact on the patient and her family [111, 112]. Therefore, it is vital that these women are followed by a multidisciplinary team, where the psychological impact of this diagnosis and its treatment can be addressed. This approach will ensure optimal, holistic care for women with GTN.

## Figures and Tables

**Table 1:** FIGO anatomic staging for gestational trophoblastic neoplasia (GTN).

Stage I	Disease confined to the uterus
Stage II	GTN extends outside of the uterus, but is limited to the genital structures (adnexa, vagina, broad ligament)
Stage III	GTN extends to the lungs, with or without known genital tract involvement
Stage IV	All other metastatic sites

FIGO: International Federation of Gynecology and Obstetrics.

**Table 2:** FIGO prognostic scoring system*.

Scores	0	1	2	4
Age (years)	≤39	≥40	—	—
Antecedent pregnancy	Mole	Abortion	Term	
Interval from index pregnancy (months)	<4	4–6	7–12	>12
Pretreatment serum hCG (IU/liter)	<1000	<10,000	<100,000	>100,000
Largest tumor size (incl, uterus)	—	3-4 cm	>5 cm	—
Site of metastases	Lung	Spleen/kidney	GI	Liver/brain
Number of metastases	—	1–4	5–8	>8
Previous failed chemotherapy			Single drug	2 or more drugs

*Format for reporting to FIGO Annual Report: in order to stage and allot a risk factor score, a patient's diagnosis is allocated to a stage as represented by a roman numeral I, II, III, and IV. This is then separated by a colon from the sum of all the actual risk factor scores, which is expressed in Arabic numerals (e.g., stage II:4, stage IV:9). This stage and score will be allotted for each patient.

FIGO: International Federation of Gynecology and Obstetrics.

**Table 3:** Treatment protocols for stage I gestational trophoblastic neoplasia (New England Trophoblastic Disease Center).

Initial	Sequential MTX/ACT-D
	Hysterectomy (with adjunctive single-agent chemotherapy)

Resistant to both single-agents	MAC
	EMACO, if MAC fails
	Hysterectomy (with adjunctive multiagent chemotherapy)
	Local uterine resection (for localized lesion, to preserve, fertility)

Followup	12 consecutive months of normal hCG levels
	Contraception mandatory

ACT-D: actinomycin D; EMACO: etoposide, methotrexate, actinomycin D, cytoxan, oncovin; MAC: methotrexate, actinomycin D, cytoxan; MTX: methotrexate.

**Table 4:** Treatment protocol for stages II and III gestational trophoblastic neoplasia (New England Trophoblastic Disease center).

*Low risk *	
Initial therapy	Sequential MTX/ACT-D
Resistant therapy	MAC or EMA/CO
	Surgery, as indicated

*High risk *	
Initial therapy	EMACO
Resistant therapy	EMAEP
	VBP
	Surgery, as indicated

Followup	12 consecutive months of undetectable hCG levels
	Contraception for 12 months

ACT-D: actinomycin D; EMACO: etoposide, methotrexate, actinomycin D, cytoxan, oncovin; EMAEP: etoposide, methotrexate, actinomycin D, carboplatin; MAC: methotrexate, actinomycin D, cytoxan; MTX: methotrexate; VBP: vinblastine, bleomyCin, carboplatin.

**Table 5:** Treatment protocol for stage IV gestational trophoblastic neoplasia (New England Trophoblastic Disease Center).

Initial	EMACO
	With brain metastases:
	Radiation
	Craniotomy for peripheral lesions
	With liver metastases:
	Embolization
	Resection to manage complications

Resistant	Salvage chemotherapy:
	EMAEP
	VBP
	Experimental protocols
	Surgery, as indicated
	Hepatic artery infusion or embolization, as indicated

Followup	Weekly hCG levels until undetectable for 3 weeks, then monthly for 24 months
	Contraception for 24 months

EMACO: etoposide, methotrexate, actinomycin D, cytoxan, oncovin; EMAEP: etoposide. methotrexate, actinomycin D, carboplatin; VBP: vinblastine, bleomycin, carboplatin.
